# Alveolar Arterial Gradient and Respiratory Index in Predicting the Outcome of COVID-19 Patients; a Retrospective Cross-Sectional Study

**DOI:** 10.22037/aaem.v10i1.1543

**Published:** 2022-04-14

**Authors:** Abhishek Singh, Kapil Dev Soni, Yudhyavir Singh, Richa Aggarwal, Vineeta Venkateswaran, Mohd Suhail Ashar, Anjan Trikha

**Affiliations:** 1 Department of Anaesthesiology, Pain Medicine & Critical Care, All India Institute of Medical Sciences, New Delhi, India.; 2 Department of Critical and Intensive Care, Jai Prakash Narayan Apex Trauma Center, All India Institute of Medical Sciences, New Delhi, India.

## Abstract

**Introduction:**

Alveolar arterial (A-a) oxygen gradient and respiratory index can be of immense help for the critical care physician in clinical decision making. This study aimed to evaluate the potential application of A-a oxygen gradient and respiratory index in predicting the survival of COVID-19 patients in intensive care unit (ICU).

**Method:**

This is a retrospective cross-sectional study involving 215 adult patients with COVID-19 disease, admitted to the ICU between 1^st^ April 2020 and 30 June 2021. Details regarding demographic variables, comorbidities, laboratory and arterial blood gas (ABG) findings were recorded. Alveolar-arterial gradient and respiratory index were calculated and tested as predictors of survival.

**Result:**

The mean age of the patients was 51.92 years (65.6 % male). Hypertension was the most common comorbidity and oxygen via non-rebreathing mask was the most common modality used at the time of ICU admission. Mortality was 28.37% and average length of stay was 12.84 days. Patients who died were older (p=0.02), mostly male (p=0.017), had at least one comorbidity (p<0.001), and higher heart rate and respiratory rate (<0.001 and p=0.03, respectively), lower pH on arterial blood gas (ABG) (p=0.002), higher FiO2 requirement (p<0.001), and increased A-a oxygen gradient on admission compared to survivors. According to receiver operating characteristic (ROC) curve analysis, A-a oxygen gradient and respiratory index were not sensitive or specific in predicting mortality in the studied patient subset.

**Conclusion:**

A-a oxygen gradient and respiratory index calculated at time of admission to ICU in patients with COVID-19 were poor predictors of survival.

## 1. Introduction:

*Coronavirus* disease (*COVID*-*19*) has posed a significant challenge to the healthcare systems worldwide. Hospitals are overwhelmed with patients suffering from severe COVID-19 infection with arterial hypoxemia progressing to acute respiratory distress syndrome (ARDS) requiring intensive care unit (ICU) admission and invasive mechanical ventilation (1). Different studies have identified several risk factors associated with poor outcome in COVID-19 patients. Some researchers have even developed prognostic models using combinations of different risk factors with good performance (AUC>0.8), but none of these studies included respiratory index (RI) and alveolar-arterial (A-a) oxygen gradient amongst the studied predictors (2-5). The paO2/ FiO2 (P/F) ratio is widely used in clinical practice to predict disease outcome in critically ill patients but RI and A-a gradient has not been in much use. P/F ratio is a good measure of lung dysfunction as depicted by Berlin criteria in ARDS (6). The elevated A-a oxygen gradient associated with hypoxemia is also a good indicator of ventilation/perfusion mismatch and intra-pulmonary shunting (7). An editorial by Tobin et al. titled “Basing Respiratory Management of COVID-19 on Physiological Principles” has explained the importance of calculating A-a oxygen gradient in COVID-19 patients. The text states that A-a oxygen gradient was more precisely able to appraise the pathophysiological basis of hypoxemia compared to the P/F ratio (8). A-a oxygen gradient can be easily derived from arterial oxygen and carbon dioxide pressures while RI can be derived from A-a gradient and arterial oxygen. Previous studies have shown the application of A-a gradient in patients with community-acquired pneumonia as an indicator of disease severity and outcome (9, 10). Therefore, in the present study, we investigated whether A-a oxygen gradient and RI could predict outcome in COVID-19 patients admitted to intensive care unit. 

## 2. Methods:

***2.1. Study design and*** ***setting***

The study was conducted in a dedicated COVID-19 care centre of All India Institute of Medical Sciences, New Delhi. The retrospective data presented in the study is part of a project that was approved by the institute ethics committee (IEC-291/17.04.2020). The requirement for written informed consent from individual patients was waived due to the retrospective observational nature of the study. 

***2.2. Participants***

The study included all adult patients (age > 18 years) with confirmed COVID-19 infection (Reverse-transcription polymerase-chain reaction (RT-PCR)-confirmed report for SARS-CoV2), admitted to the ICU between 1^st^ April 2020 and 30 June 2021 with available electronic medical records. All patients with incomplete or missing data with respect to demographics, comorbidities, vital signs, laboratory findings, arterial blood gas, and outcome were excluded from the study.

***2.3. Data gathering***

Data were retrospectively collected using medical records and computerized patient record system. Data collected included demographics (age, sex), vital parameters (heart rate, respiratory rate, blood pressure, temperature), comorbidities, type of oxygen support on admission, FiO2 used, spO2 (oxygen saturation), arterial blood gas (ABG) analysis, blood cell count, renal function tests, liver function test, duration of hospital stay, and outcome.

***2.4. Definitions***

- A-a gradient was calculated using the following formula:

AaDO2 = [(FiO2) (Atmospheric pressure − H2O pressure) − (PaCO2/R)] − PaO2

Standard values of atmospheric pressure (760 mmHg), H2O pressure (47 mmHg) and R (0.80) were considered for calculating the A-a Gradient. The A-a oxygen gradient and age- adjusted A-a oxygen gradient were also calculated for each patient. Since A-a oxygen gradient is dependent on age, the age-adjusted A-a gradient might be more accurate for detecting disease severity. The age-adjusted A-a oxygen gradient was calculated by subtracting the expected A-a oxygen gradient for age from the measured A-a oxygen gradient. The expected A-a oxygen gradient for age was derived using the following formula (Age/4) + 4. 

Respiratory index was calculated using the following formula: RI= A-a oxygen gradient /PaO2.

***2.5. Outcome***

The primary outcome of our study was to determine whether A-a oxygen gradient and RI at the time of admission to ICU could predict the outcome of COVID-19 patients in the ICU.

***2.6. Statistical analysis***

Continuous variables were expressed as mean ± standard deviation ( and categorical variables as number (percentage). Group comparison was performed using independent t-tests or Chi-square tests. A receiver operating characteristic (ROC) curve was made and area under the curve (AUC) was calculated to assess an optimal cut-off value of the A-a oxygen gradient and RI. A p-value less than 0.05 was considered statistically significant.

## 3. Results:

***3.1. Baseline characteristics of studied patients***

A total of 300 patients with confirmed COVID-19 infection admitted to the ICU during the study period were screened for possible inclusion in the study. Two hundred eighty patients met the inclusion criteria. After excluding 65 patients due to missing or incomplete data, the final studied cohort consisted of 215 patients. These were subdivided into survivors and non-survivors as well as into mild, moderate, or severe ARDS based on P/F ratio**.**

Table 1 shows the patient’s demographics, comorbidities, vital signs, laboratory parameters, ABG values, A-a oxygen gradient, and RI at time of admission to ICU. The mean age of the patients was 51.92 ±13.89 years (65.6% male). 114 (53.3%) cases had at least one comorbidity. Hypertension was the most common comorbidity (37.4%) followed by diabetes (26.2%), chronic kidney disease (10.3%), malignancy (5.6%), and tuberculosis (1.9%). Mean vital signs on admission, namely heart rate, systolic blood pressure, diastolic blood pressure, respiratory rate, and oxygen saturation were 99.46 per minute, 127.97 mmHg, 78.10 mmHg, 24.26 per minute, and 94.89%, respectively. Laboratory parameters were grossly normal except for slightly deranged renal and liver functions. 

Table 2 shows the modalities of oxygen therapy for studied cases. The most common oxygen therapy modality used at the time of ICU admission was non-rebreathing mask (67.4%) followed by high-flow nasal cannula (HFNC) (10.7%), mechanical ventilation (9.8%), oxygen via face mask (5.6%), and non-invasive ventilation (NIV) (5.6%). Arterial blood gas on admission showed a mean pH of 7.39, pCO2 36.42 mmHg, pO2 97.06 mmHg, P/F ratio 154.98, A-a gradient 314.07 mmHg, normal gradient for age 16.98 mmHg, age-adjusted A-a oxygen gradient 302.43 mmHg, and RI of 4.50. Mortality rate was 28.37% and average length of stay was 12.84 days.

***3.2. Predictors of mortality***

Subgroup analysis amongst survivors and non-survivors showed that non-survivors were older (p = 0.02), mostly male (p = 0.017), had at least one comorbidity (p = 0.001), and had higher heart rate and respiratory rate (p = 0.001 and p = 0.03, respectively) compared to survivors. They also had higher total leucocyte counts, higher values of serum urea, blood creatinine, bilirubin, liver enzymes and INR, and lower values of serum protein and albumin compared to survivors (Table 1). 

The analysis of ABG and oxygen therapy on admission showed that that non-survivors had lower pH (p = 0.002), higher FiO2 requirement (p = 0.001), and increased A-a oxygen gradient (including calculated, normal gradient for age, and age-adjusted A-a oxygen gradient) on admission. 

A reduction in Pao2 and increased A-a oxygen gradient were associated with presence of severe COVID-19. However, there was no significant difference in the RI between survivors and non-survivors. 

After stratifying the patients into mild, moderate and severe ARDS based on P/F ratio, we found that an increased A-a gradient was associated with increased severity of ARDS (p = 0.001). Patients with severe ARDS had higher oxygen requirement, increased A-a oxygen gradient, lower PaO2, lower PaCO2, and higher RI compared to patients with mild or moderate ARDS.

***3.3. Predictive value of A-a gradient and RI***

**Figure 1:**
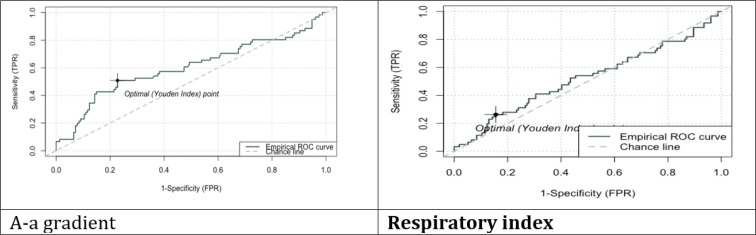
Receiver operating characteristic (ROC) curve of alveolar arterial gradient (best cut-off = 0.28; area under the curve (AUC) = 0.602) and respiratory index (best cut-off = 0.28; AUC = 0.522) in predicting the mortality of COVID-19 cases

Figure 1 and table 3 show the ROC curves of A-a oxygen gradient and RI in predicting the mortality of COVID-19 cases. The area under the curve (AUC), sensitivity, specificity, positive predictive value, and negative predictive value of A-a gradient in this regard were 0.602, 57%, 57%, 35%, and 77%, respectively. In addition, the AUC, sensitivity, specificity, positive predictive value, and negative predictive value of RI in predicting the outcome of COVID-19 cases were 0.522, 54%, 48%, 29%, and 73%, respectively.

**Table 1:** Demographic and clinical characteristics of COVID-19 patients

**Parameter **	**Overall (n-215)**	**Survivor (n-154)**	**Dead (n=61)**	**P **
**Age (Year) **	51.92 ±17.89	49.52 ±17.49	58.05 ±17.55	0.022
**Gender**				
Male	141 (65.6)	93 (60.4)	48 (78.7)	0.017
Female	74 (34.4)	61(39.6)	13(21.3)	
**Length of stay **				
Mean ± SD	12.84 ± 9.31	13.50 ± 9.36	11.16 ± 9.05	0.097
**Comorbidities **				
Any type	114 (53.3)	71(46.4)	43 (70.5)	0.002
HTN	80 (37.4)	52 (34)	28 (45.9)	0.142
DM	56 (26.2)	35 (22.9)	21 (34.4)	O.118
CKD	22 (10.3)	13 (8.5)	9 (14.8)	0.266
Malignancy	12 (5.6)	3 (1.9)	9 (14.8)	0.001
TB	4 (1.9)	2 (1.3)	2 (3.3)	0.68
**Vital signs**				
Fever °c	25 (11.6)	19 (13.4)	6 (9.8)	0.63
HR (/minutes)	99.46 ± 21.12	95.38 ± 18.71	109.43 ± 23.41	0.001
SBP (mmHg)	127.97 ± 21.89	127.36 ± 21.37	129.44 ± 23.22	0.53
DBP (mmHg)	78.10 ± 12.93	78.25 ± 12.23	77.75 ± 14.60	0.80
RR (/minute)	24.26 ± 5.62	23.64 ± 5.11	25.57 ± 6.44	0.03
SaO2 (%)	94.89 ± 6.25	95.16 ± 5.34	94.21 ± 8.14	0.32
**Laboratory Parameters**				
Hb (mg/dl)	11.00 ± 2.45	10.98 ± 2.39	11.04 ± 2.62	0.88
WBC (×10^3^)	12.03 ± 8.07	11.28 ± 8.29	13.85 ± 7.27	0.03
PLT (×10^3^)	227.80 ± 194.01	237.46 ± 214.18	204.46 ± 131.91	0.26
Urea (mg/dl)	59.81 ± 58.79	47.91 ± 35.80	89.37 ± 87.89	0.001
Creatinine(mg/dl)	1.61 ± 2.58	1.40 ± 2.09	2.14 ± 3.48	0.06
Sodium(mEq/L)	134.52 ± 9.00	133.18 ± 7.61	137.34 ± 10.94	0.003
Potassium(mmol/L)	4.23 ± 0.82	4.21 ± 0.76	4.26 ± 0.95	0.732
Bilirubin(mg/dl)	1.32 ± 3.09	1.03 ± 1.51	2.06 ± 5.20	0.02
SGOT(U/L)	173.54 ± 1122.61	71.16 ± 146.95	414.14 ± 2033.93	0.04
SGPT(U/L)	89.66 ± 210.40	58.38 ± 105.16	163.16 ± 340.57	0.001
TP(g/dl)	6.28 ± 0.99	6.36 ± 0.94	6.08 ± 1.10	0.064
Albumin(g/dl)	2.83 ± 0.70	2.89 ± 0.70	2.69 ± 0.67	0.062
GB(g/dl)	3.44 ± 0.72	3.46 ± 0.69	3.37 ± 0.77	0.419
ALP(U/L)	132.57 ± 239.35	137.66 ± 281.32	120.68 ± 81.06	0.647
INR	1.29 ± 0.60	1.21 ± 0.63	1.42 ± 0.53	0.04
aPTT (second)	33.45 ± 26.22	33.88 ± 31.66	32.62 ± 9.34	0.774
**ABG ** **parameters**				
pH	7.39 ± 0.13	7.41 ± 0.09	7.35 ± 0.18	0.002
pO2(mmHg)	97.06 ± 68.64	94.71 ± 66.58	102.99 ± 73.82	0.427
pCo2(mmHg)	36.42 ± 9.87	36.12 ± 9.37	37.17 ± 11.06	0.483
Fio2	0.64 ± 0.11	0.62 ± 0.10	0.69 ± 0.13	0.001
P/F Ratio	151.99 ± 97.27	150.60 ± 91.97	155.51 ± 110.25	0.739
A-a Gradient(mmHg)	316.72 ± 91.84	307.85 ± 79.88	339.13 ± 114.46	0.024
Normal	16.98 ± 4.47	16.38 ± 4.37	18.51 ± 4.39	0.002
Age-adjusted	301.14 ± 89.54	291.48 ± 80.28	326.16 ± 106.75	0.011
RI	4.50 ± 2.52	4.41 ± 2.33	4.73 ± 2.96	0.40

Data are presents as mean ± standard deviation (SD) or number (%). HTN: hypertension; DM: diabetes mellitus; CKD: chronic kidney disease; TB: tuberculosis; HR: heart rate; SBP: systolic blood pressure; DBP: diastolic blood pressure; RR: respiratory rate; Hb: haemoglobin; WBC: white blood cell; PLT: platelet; SGOT: serum glutamic-oxalo acetic transaminase; SGPT: serum glutamic-pyruvic transaminase; TP: total protein; GB: globulin; ALP: alkaline phosphatase; INR: international normalized ratio; aPTT: activated partial thromboplastin time; Fio2: fraction of inspired oxygen; P/F ratio: ratio of arterial partial pressure of oxygen to inspired fractional concentration of oxygen; A-a gradient: alveolar arterial oxygen gradient; RI: respiratory index.

**Table 2:** Comparing the survival rate based on different respiratory supports

**Mode **	**Overall (n-215)**	**Survived (n-154)**	**Died (n-61)**	**P value**
Face mask	12 (5.6)	10 (6.5)	2 (3.3)	0.001
NRBM	145 (67.4)	120 (78.4)	25 (41.7)	
HFNC	23 (10.7)	14 (9.2)	9 (15.0)	
NIV	12 (5.6)	6 (3.9)	6 (10.0)	
MV	21 (9.8)	3 (2.0)	18 (30.0)	

Data are presented as number (%). NRBM: non-rebreathing mask; HFNC: high-flow nasal cannula; NIV: non-invasive ventilation; MV: mechanical ventilation.

**Table 3:** Screening performance characteristics of A-a gradient and respiratory index in predicting the mortality of COVID-19 patients

**Character**	**A-a gradient**		**Respiratory index**
	**Normal **	**Age-adjusted**	
**Sensitivity**	0.57	0.52	0.54
**Specificity**	0.57	0.62	0.48
**PPV**	0.35	0.35	0.29
**NPV**	0.77	0.77	0.73
**PLR**	1.32	1.36	1.03
**NLR**	0.75	0.77	0.95
**AUC (95%CI)**	0.63(0.55-0.72)	0.58(0.48-0.67)	0.52(0.43-0.61)

Data are presented with 95% confidence interval in 0.28 cut-off point. A-a gradient- alveolar arterial oxygen gradient; PPV: positive predictive value; NPV: negative predictive value; PLR: positive likelihood ratio; NLR: negative likelihood ratio; AUC: area under the receiver operating characteristic (ROC) curve.

## 4. Discussion:

The present study found that A-a oxygen gradient and RI on admission are poor predictors for outcome in COVID-19 patients admitted to the ICU. The A-a oxygen gradient is the measure of difference between oxygen level in the alveoli and arterial blood (8). It helps differentiate between hypoxemia due to dysfunction of alveolar capillary unit, in which A-a oxygen gradient is raised, or due to pump failure, in which the gradient is normal. Data on prognostic utility of A-a oxygen gradient and RI in patients with COVID-19 are scarce at present. Because of the effect of COVID-19 disease on pulmonary gas exchange, shunting, and ventilation/perfusion ratio, we decided to evaluate the efficacy of A-a oxygen gradient and RI in guiding critical care clinicians. Various biomarkers have been shown to correlate with the clinical outcome in COVID-19 patients (11, 12). However, most of them are time- and resource- intensive, while ABG analysis is easily available in all critical care units and gives additional useful information on acid-base status, oxygenation, and ventilation status, amongst others.

The relationship between A-a gradient and outcome has already been demonstrated in patients with community acquired pneumonia (9, 10, 13). Avci et al. showed that A-a oxygen gradients were robust predictors of 30-day mortality in patients with community acquired pneumonia and demonstrated even better performance than inflammatory markers like CRP or scores like PSI or CURB-65 (13).

Few studies have been published evaluating the predictive value of A-a oxygen gradient on admission as a tool for diagnosis, triage, clinical decision-making, or predicting outcome. Gabrielli et al., in their retrospective study on relationship between Alveolar-arterial oxygen gradient, mortality, and admission to intensive care unit in severe COVID-19-related pneumonia, found that A-a O2 gradient, which was calculated for patients with severe COVID-19 on arrival to emergency department, was able to predict early admission to ICU, but not mortality (14). Similarly in our study, A-a gradient and RI were neither sensitive nor specific predictors of mortality in COVID-19 patients in the ICU. Gupta et al., in their retrospective cohort study, found that Alveolar-arterial oxygen gradient was a good predictor of mortality in COVID-19 patients started on non-invasive ventilation (NIV) for increasing respiratory distress (15). It could be due to inclusion of patients with severe disease requiring NIV, while our study included patients receiving oxygen from face mask to ventilator. Carlino et al., in their observational study, demonstrated that patients admitted to ICU have higher A-a gradients than non-ICU patients, and that A-a gradient has good accuracy (AUC of 0.952) in predicting ICU admission in patients with COVID-19 (16). De roos MP et al., in their retrospective analysis, showed that low dose chest computed tomography and A-a gradient may serve as rapid and accurate tools to diagnose COVID-19 pneumonia and to select mildly symptomatic patients in need for hospitalization (17). Secco et al., in their study, showed that A-a gradient and lung ultrasound are effective tools for bedside risk stratification of COVID-19 patients when P/F ratio and clinical manifestations do not indicate severe lung dysfunction (18). In the present study, we also found that the predictors of poor prognosis were advanced age, male gender, presence of comorbidities, malignancy, raised heart rate and respiratory rate, increased total leucocyte counts, altered liver and renal function, lower pH, higher oxygen requirement, and increased A-a gradient. The reason for such result is multifactorial. It can be due to interplay of multiple factors like hypovolemia due to fever and reduced fluid intake, right or left ventricular dysfunction due to invasive mechanical ventilation with high positive end-expiratory pressure, myocarditis, pulmonary embolism, and vasodilation and increased capillary leak due to sepsis and cytokine storm. Other reasons can be changing guidelines with respect to steroid therapy, antiviral therapy, and self proning, which may have affected the outcome.

## 5. Limitation

Our study had several limitations. Firstly, it was a retrospective single centre study with a number of missing variables and inevitable bias in identifying and recruiting patients. Secondly, causes of raised A-a oxygen gradient unrelated to COVID-19, for instance pulmonary embolism, were not evaluated. Thirdly, our study had a relatively small sample size of 215 patients, hence external validation of the findings is needed to warrant its clinical utility.

## 6. Conclusion:

Our study demonstrated that A-a oxygen gradient and respiratory index are not effective in predicting mortality among COVID-19 patients in the ICU. However, our findings need confirmation in well-designed studies with large numbers of patients.

## 7. Declarations:

### 7.1. Acknowledgment

None.

### 7.2. Authors’ contributions

A.S, K.D.S, R.A, Y.S, M.S.A , V.V, A.T -Contributed to conception, study design, and data collection and evaluation. A.S and KDS- Contributed to statistical analysis, and interpretation of data A.S, Y.S, AT; Drafted the manuscript, which was revised by K.D.S. All authors read and approved the final manuscript.

### 7.3. Conflict of interest

None.

### 7.4. Funding and supports

None
